# The Prevalence of Depression and Its Associated Risk Factors Among Government Primary School Teachers in Dammam, Khobar, and Qatif (2019-2021): A Cross-Sectional Study

**DOI:** 10.7759/cureus.36271

**Published:** 2023-03-16

**Authors:** Muayad Y AL Awwas, Omar S Alqasem, Hussain M Alhussain, Areej M Alqahtani

**Affiliations:** 1 Family Medicine, Alahsa Health Cluster, Alahsa, SAU; 2 Family Medicine, King Fahad Military Medical Complex Hospital, Ministry of Defense, Dhahran, SAU; 3 Family Medicine, Eastern Health Cluster, Dammam, SAU; 4 Family Medicine, Dammam Health Network, Eastern Health Cluster, Dammam, SAU

**Keywords:** dammam, saudi, mental health, teacher, depression

## Abstract

Introduction: In Saudi Arabia, 34% of Saudis have been diagnosed with a mental health illness at some point in their lives, with depression making up to 6% of the population. Teachers' mental health is a major problem across the world and has an impact on their students. This study is an attempt to investigate the prevalence of depression and its severity and associated sociodemographic and occupational risk factors among government primary school teachers in Dammam, Khobar, and Qatif.

Methods: This is a cross-sectional study. The research tool used to conduct this study is an electronically administered Arabic-language questionnaire distributed randomly to all government primary school teachers in Dammam, Khobar, and Qatif. The numbers of participating teachers are 358,242 males and 116 females.

Results: By using the Patient Health Questionnaire 9 (PHQ9) scale, it was found that 36.6% suffer from mild depression, 30.4% suffer from moderate to moderately severe depression, and 11.2% suffer from severe depression. The obtained results indicated that there is an association between the variable of the prevalence of depression and some sociodemographic factors such as physical or psychosocial abuse, and the occupational characteristic variables such as teaching more than three or more subjects and bad relationship with the school administration.

Conclusion: More studies are needed to address the mental health issues among school teachers in Saudi Arabia.

## Introduction

Education enhances one's learning, abilities, character, and mindset; it also develops a person's personality and attitude. Today's educational system aims to prepare students not only for future careers but also for physical and social well-being. Although teaching is seen as a noble vocation in many countries, teachers are subjected to a variety of stressful conditions, which can result in a variety of mental disorders [1]. Teachers in primary school have enormous responsibilities and challenging tasks for their students to establish and translate most of the basic scientific and theoretical knowledge. They are also responsible for raising the children, organizing classes and activities, and managing extra work in case of teacher absence, which requires a lot of energy to deal with such situations [2].

School teachers are reported to spend half of their lives on work-related duties [3]. Stress is considered to be a risk factor for depression among workers in different professions [4,5]. Many studies have shown a relationship between depression and work stress [6]. Many different studies that were conducted in several regions of the world have shown a high level of stress among teachers; 22% of teachers in Germany believe that their job is stressful as was shown in the study that was carried out in Germany in 2007 [7]. Another study was conducted in Egypt in 2017, and the result was that 100% of Egyptian teachers were stressed [8]. Furthermore, burnout is a natural outcome of a high-stress profession, and burnout in teachers can have a detrimental impact on students and the quality of teaching [9].

Depression is one of the most common psychiatric problems today, impacting social life and individuals in their long-life period, as well as their satisfaction, functioning, and interest in life and work [10]. Depression has been identified as one of the primary causes of disability globally by the World Health Organization (WHO) [11] with more than 300 million depressed people around the world [12]. Generally, depression can affect teachers' emotions and performance and daily activity [13,14]. In Saudi Arabia, 34% of Saudis are diagnosed at least once in their life with mental health disorders [15]; the prevalence of depression is about 6%, affecting females more than males [15]. The Saudi National Mental Health Survey reports found an association between mental health conditions and educated persons [15].

Teachers' mental health is a major problem across the world. Thus, numerous studies have been conducted to estimate depression among teachers; one of these studies was conducted on Mexican teachers in 2018 and found that 16% of them had severe depression [16]. However, there is limited data on depression among school teachers in the Middle East. One of the few pieces of research on Egyptian teachers done in 2017 revealed that depression affects 23% of them [8]. Another research, conducted in 2008 among secondary school teachers in Saudi Arabia's Asser area, found that 25% of teachers were depressed [17]. Many risk factors have been linked to depression, and chronic illness has been shown to aggravate depressive symptoms [18], as well as smoking and medication used such as analgesic, corticosteroid, and calcium channel blocker [19,20]. A major life event that is stressful, such as a family member's death or a loved one or the loss of a job, has also been closely linked to the beginning of depressive symptoms [21], and being subjected to verbal abuse at work is strongly linked to job stress [22]. The aim of this study is to measure the prevalence and severity of depression among government primary school teachers in Dammam, Khobar, and Qatif and also to study the association between the sociodemographic and the occupational characteristics with the prevalence of depression.

## Materials and methods

A cross-sectional study was carried out on a sample of government primary school teachers. To assess levels of depression among teachers, the validated Arabic version of the Patient Health Questionnaire 9 (PHQ9) [23] was used in the questionnaire. Its reliability was found to be 0.857 [24]. It consists of three sections that include (independent variables) sociodemographic information such as gender, age, marital status, number of children, occupational characteristics such as the place of school, educational qualification, income, nature of the job, stage of classes handled, number of subjects and classes, years of experience, and gender of students. The third part (dependent variable) consists of a depression scale. It is a nine-item scale; each item was scored from 0 to 3 according to the symptom severity where the total score ranged from 0 to 27. The cutoff points used to determine depression levels were as follows: minimal or no depression if the score ranged from 1 to 4, mild depression if ranged from 4 to 9, moderate to moderately severe depression if ranged from 10 to 19, and severe depression if ranged from 20 to 27 [23].

The study was conducted in the time frame from October 2019 to September 2021. It was composed of male and female teachers. The electronic questionnaire was sent to the general directors of Dammam, Khobar, and Qatif educational sectors, and then, it was distributed to all male and female government primary school teachers. The sample included all male and female government primary school teachers working in Dammam, Khobar, and Qatif, who were full-time permanent employees. Teachers who are working in special or private schools were excluded. The data was loaded into a computer and analyzed with the Statistical Package for Social Sciences (SPSS) program version 25 (IBM SPSS Statistics, Armonk, NY). The data has been coded and divided into numbers and percentages; then, the chi-square test was applied to test the association between the available variables. A P value of <0.01 was considered statically significant. Using the nesting method is more appropriate for such a study, and this can be considered one of the limitations.

## Results

A total of 358 teachers participated, which included 242 male participants (67.6%) against 116 female participants (32.4%). The mean age of the participating teachers is 42 with the minimum age being 22 and the maximum being 74. The mean number of the weekly lesson is 20 classes. The study sample has shown that most of the participants are married with more than one child. As Table 1 indicated, 28.7% of teachers (102 teachers) suffered from chronic diseases; also, 30.7% are in continuous drug use. Of the participants, 22.1% experienced psychological trauma at work, while 27% of them has a history of psychological events such as bereavement in the past. Table 1 shows all the sociodemographic features of the participants.

**Table 1:** Sociodemographic characteristics of the participating teachers

		Count	%
Sex	Male	242	67.6%
	Female	116	32.4%
Marital status	Single	22	6.1%
	Married	319	89.1%
	Divorced	11	3.1%
	Widow	6	1.7%
Number of kids	1	46	12.8%
	2	81	22.6%
	3 and more	231	64.5%
Chronic disease	Yes	102	28.7%
	No	254	71.3%
Continuous drug use	Yes	109	30.7%
	No	246	69.3%
History of physical or psychological trauma at work	Yes	78	22.1%
	No	275	77.9%
History of psychological event	Yes	97	27.5%
	No	256	72.5%
Smoking	Yes	70	19.7%
	No	286	80.3%

Table 2 shows that most of the teachers are qualified with bachelor's degree. Seventy-six percent of them are teaching without any other responsibilities, while 24% of them are teaching and fulfilling administrative tasks. Classes with male students represent 67.3% while females 28.8%; mixed classes represent 3.9% of the study sample. Also, 41% of the teachers have a good relationship with their administration.

**Table 2:** Occupational characteristics of the participating teachers SAR: Saudi Arabian Riyal

		Count	%
Academic degree	Diploma	10	2.8%
	Bachelor's	328	91.6%
	Master's	19	5.3%
	PhD	1	0.3%
Job nature	Teaching only	272	76.0%
	Teaching and administration	86	24.0%
Class level	Lower grades (1-3)	120	33.5%
	Mix grades (1-6)	90	25.1%
	Higher grades (4-6)	148	41.3%
Student's gender	Male	241	67.3%
	Female	103	28.8%
	Mix	14	3.9%
Number of subjects	1	99	27.7%
	2	77	21.5%
	3 and more	182	50.8%
Teaching experience in years	Less than two	10	2.8%
	2-5	28	7.8%
	5-10	54	15.1%
	10-20	120	33.5%
	More than 20	146	40.8%
Salary	5,000-10,000 SAR	1	0.3%
	10,000-15,000 SAR	218	62.6%
	15,000-20,000 SAR	121	34.8%
	>20,000 SAR	8	2.3%
Evaluation	Below good	0	0.0%
	Good	2	0.6%
	Very good	22	6.2%
	Excellent	331	93.2%
Relationship with school administration	Good	145	41.0%
	Normal	189	53.4%
	Bad	20	5.6%

When identifying the prevalence and severity of depression among public primary school teachers in Dammam, Khobar, and Qatif using the PHQ9 scale, it was found that 21.8% have no depression, 36.6% suffer from mild depression, 30.4% suffer from moderate to moderately severe depression, and 11.2% suffer from severe depression.

Table 3 clearly shows that there is a relationship between the variables (gender, history of physical trauma at work, and history of psychosocial event) and the level of depression (P<0.01). Table 4 shows that there is significant relationship between the level of depression and some occupational characteristics (student's gender, number of subjects, and relationship with school administration).

**Table 3:** Association between demographic characteristics and the level of depression

		Depression scale 2								P value
		No depression		Mild depression		Moderate to moderately severe depression		Severe depression		
		N	%	N	%	N	%	N	%	
Sex	Male	70	28.9%	98	40.5%	59	24.4%	15	6.2%	<0.001
	Female	8	6.9%	33	28.4%	50	43.1%	25	21.6%	
Marital status	Single	4	18.2%	6	27.3%	10	45.5%	2	9.1%	0.164
	Married	74	23.2%	119	37.3%	92	28.8%	34	10.7%	
	Divorced	0	0.0%	5	45.5%	3	27.3%	3	27.3%	
	Widow	0	0.0%	1	16.7%	4	66.7%	1	16.7%	
Number of kids	1	10	21.7%	16	34.8%	15	32.6%	5	10.9%	0.297
	2	10	12.3%	37	45.7%	26	32.1%	8	9.9%	
	3 and more	58	25.1%	78	33.8%	68	29.4%	27	11.7%	
Chronic disease	Yes	18	17.6%	35	34.3%	34	33.3%	15	14.7%	0.343
	No	60	23.6%	95	37.4%	74	29.1%	25	9.8%	
Continuous drug use	Yes	15	13.8%	42	38.5%	37	33.9%	15	13.8%	0.085
	No	63	25.6%	88	35.8%	70	28.5%	25	10.2%	
History of physical or psychological trauma	Yes	4	5.1%	26	33.3%	32	41.0%	16	20.5%	<0.001
	No	74	26.9%	102	37.1%	75	27.3%	24	8.7%	
History of psychological trauma	Yes	13	13.4%	28	28.9%	42	43.3%	14	14.4%	0.001
	No	65	25.4%	101	39.5%	65	25.4%	25	9.8%	
Smoking	Yes	9	12.9%	28	40.0%	26	37.1%	7	10.0%	0.171
	No	69	24.1%	102	35.7%	82	28.7%	33	11.5%	

**Table 4:** Association between occupational characteristics and level of depression SAR: Saudi Arabian Riyal

		Depression scale 2								P value (chi-square)
		No depression		Mild depression		Moderate to moderately severe depression		Severe depression		
		N	%	N	%	N	%	N	%	
Academic degree	Diploma	3	30.0%	4	40.0%	3	30.0%	0	0.0%	0.731
	Bachelor's	70	21.3%	120	36.6%	99	30.2%	39	11.9%	
	Master's	4	21.1%	7	36.8%	7	36.8%	1	5.3%	
	PhD	1	100.0%	0	0.0%	0	0.0%	0	0.0%	
Job nature	Teaching only	64	23.5%	96	35.3%	84	30.9%	28	10.3%	0.399
	Teaching and administration	14	16.3%	35	40.7%	25	29.1%	12	14.0%	
Class level	Lower grades (1-3)	27	22.5%	46	38.3%	35	29.2%	12	10.0%	0.308
	Mix grades (1-6)	17	18.9%	40	44.4%	21	23.3%	12	13.3%	
	Higher grades (4-6)	34	23.0%	45	30.4%	53	35.8%	16	10.8%	
Student's gender	Male	70	29.0%	98	40.7%	59	24.5%	14	5.8%	<0.001
	Female	7	6.8%	29	28.2%	44	42.7%	23	22.3%	
	Mix	1	7.1%	4	28.6%	6	42.9%	3	21.4%	
Number of subjects	1	17	17.2%	46	46.5%	30	30.3%	6	6.1%	0.020
	2	25	32.5%	19	24.7%	25	32.5%	8	10.4%	
	3 and more	36	19.8%	66	36.3%	54	29.7%	26	14.3%	
Teaching experience in years	Less than two	2	20.0%	2	20.0%	3	30.0%	3	30.0%	0.063
	2-5	5	17.9%	9	32.1%	13	46.4%	1	3.6%	
	5-10	5	9.3%	27	50.0%	15	27.8%	7	13.0%	
	10-20	27	22.5%	46	38.3%	38	31.7%	9	7.5%	
	More than 20	39	26.7%	47	32.2%	40	27.4%	20	13.7%	
Salary	5,000-10,000 SAR	0	0.0%	1	100.0%	0	0.0%	0	0.0%	0.185
	10,000-15,000 SAR	38	17.4%	78	35.8%	72	33.0%	30	13.8%	
	15,000-20,000 SAR	35	28.9%	44	36.4%	32	26.4%	10	8.3%	
	>20,000 SAR	1	12.5%	5	62.5%	2	25.0%	0	0.0%	
Evaluation	Below good	0	0.0%	0	0.0%	0	0.0%	0	0.0%	0.319
	Good	0	0.0%	2	100.0%	0	0.0%	0	0.0%	
	Very good	3	13.6%	8	36.4%	6	27.3%	5	22.7%	
	Excellent	75	22.7%	120	36.3%	101	30.5%	35	10.6%	
Relationship with school administration	Good	45	31.0%	53	36.6%	36	24.8%	11	7.6%	0.004
	Normal	32	16.9%	71	37.6%	61	32.3%	25	13.2%	
	Bad	1	5.0%	5	25.0%	11	55.0%	3	15.0%	

## Discussion

The present study aimed to estimate the prevalence of depression and its associated risk factors among government primary school teachers in Dammam, Khobar, and Qatif, in the eastern province of Saudi Arabia. The study showed that 36.6% of the participants suffer from mild depression, 30.4% suffer from moderate to moderately severe depression, and 11.2% suffer from severe depression, which is considered to be a high prevalence compared to the prevalence of depression among people in Saudi Arabia, which was only 6% [15]. Also, this finding is higher than one of the studies, which has been conducted among school teachers in Aseer city, Saudi Arabia, in 2008 [19], which showed that 25% of secondary school teachers in Aseer city were depressed, but there was no categorization or estimation of the level of depression. In Egypt, Desouky and Allam (2017) found that depression affects 23% of Egyptian teachers [8]. Another study conducted in Mexico revealed that 16% of Mexican teachers are severely depressed [17], which is higher than the finding in the present study.

The data from the present study has been collected during the Covid-19 pandemic; several studies found that there is a relationship between the Covid-19 pandemic and the increased prevalence of depression among teachers. This can be one of the reasons for the high prevalence of depression among teachers in this study. Regarding the demographic characteristics of the participating teachers, there is a high prevalence of moderate to moderately severe and severe depression among females, which correlates with the finding in the Saudi National Mental Health Survey that stated that depression is more common among females [15]. There is a relationship between a history of physical or psychological trauma or abuse at work and depression; such findings are in the line with the Korean study by Oh and Kim (2015) [22]. Chronic disease and smoking are well-known risk factors for depression [19,21], but there was no relationship between them and depression in the present study. Regarding the occupational characteristics of the participating teachers, in the present work, there was an association between high numbers of teaching subjects and depression; this might be related to the high workload. The same association was found in the literature. Also, depression is lower in teachers who report good and normal relationship with the administration; this finding is similar to participants in the study that was carried out in Spain in 2007 [25]. One of the limitations of this study is the difficulty and misunderstanding of the questions that the participants faced during filling out the questionnaire, and this might lead to the inaccuracy of the entered data. Also, for the final diagnosis of depression, there is a need for a full psychiatric evaluation.

## Conclusions

The prevalence of depression among public primary school teachers in Dammam, Khobar, and Qatif is 78.2%, with 11.2 of them suffering from moderate to moderately severe depression. The study showed that there is a significant relationship between depression and participants with a history of physical or psychological abuse at work. Also, there is a significant relation between depression and participants with three or more teaching subjects, as well as participants with a bad relationship with their administrations at schools. The study recommends that there is a need for more studies in order to establish and recognize the mental health problem and in order to implant and apply the needed changes.
